# Angina in 2022: Current Perspectives

**DOI:** 10.3390/jcm11236891

**Published:** 2022-11-22

**Authors:** Roberto Manfredi, Monica Verdoia, Paolo Compagnucci, Alessandro Barbarossa, Giulia Stronati, Michela Casella, Antonio Dello Russo, Federico Guerra, Giuseppe Ciliberti

**Affiliations:** 1 Cardiology and Arrhythmology Clinic, University Hospital “Ospedali Riuniti”, 60126 Ancona, Italy; 2 Division of Cardiology Ospedale degli Infermi, ASL, 13875 Biella, Italy; 3 Department of Biomedical Sciences and Public Health, Marche Polytechnic University, 60126 Ancona, Italy

**Keywords:** stable angina, coronary artery disease, nitrates, beta-blockers, calcium channel blockers

## Abstract

Angina is the main symptom of ischemic heart disease; mirroring a mismatch between oxygen supply and demand. Epicardial coronary stenoses are only responsible for nearly half of the patients presenting with angina; whereas in several cases; symptoms may underlie coronary vasomotor disorders; such as microvascular dysfunction or epicardial spasm. Various medications have been proven to improve the prognosis and quality of life; representing the treatment of choice in stable angina and leaving revascularization only in particular coronary anatomies or poorly controlled symptoms despite optimal medical therapy. Antianginal medications aim to reduce the oxygen supply-demand mismatch and are generally effective in improving symptoms; quality of life; effort tolerance and time to ischemia onset and may improve prognosis in selected populations. Since antianginal medications have different mechanisms of action and side effects; their use should be tailored according to patient history and potential drug-drug interactions. Angina with non-obstructed coronary arteries patients should be phenotyped with invasive assessment and treated accordingly. Patients with refractory angina represent a higher-risk population in which some therapeutic options are available to reduce symptoms and improve quality of life; but robust data from large randomized controlled trials are still lacking.

## 1. Introduction

Cardiovascular disease is the leading cause of death worldwide and ischemic heart disease is responsible for approximately half of these deaths [1,2]. Among its clinical manifestations, acute coronary syndromes typically need urgency or emergency management [3], while chronic coronary syndromes can be usually managed with an outpatient approach for both diagnosis and treatment, in absence of identified high-risk features [4]. Patients with stable angina pectoris have a 3 to 4% annual incidence of myocardial infarction and death and the principal therapies available (lifestyle modifications, medications, percutaneous coronary intervention, and coronary artery bypass grafting) have the primary aim of reducing the risk of death, myocardial infarction and stroke and improve quality of life by reducing symptoms [5].

## 2. Pathophysiology of Angina Pectoris

Angina pectoris is caused by episodes of myocardial ischemia provoked by an imbalance in myocardial oxygen supply-demand, which may be due either to an increase in oxygen demand (depending on wall tension, i.e., intraventricular pressure, left ventricular radius—and volume—and wall thickness; heart rate and myocardial contractility) or a decrease in oxygen supply (reduction in coronary blood flow, anaemia and other causes reducing oxygen-carrying capacity of the blood).

Angina typically occurs when oxygen demand increases in presence of a reduced coronary low reserve, often due to an atherosclerotic coronary plaque obstructing the vessel lumen. Some patients may report symptoms and have instrumental signs of ischemia—without angiographically-significant coronary lesions—due to anomalies in the coronary circulation (vasospastic angina, coronary microvascular dysfunction) [6,7]. Abrupt reduction or interruption of blood flow, typically caused by plaque rupture/erosion and coronary thrombosis is the typical mechanism involved in acute coronary syndromes [8].

Therefore, drugs that reduce the progression of atherosclerosis and prevent plaque remodelling are recommended in coronary artery disease (CAD) patients to reduce the risk of myocardial infarction (MI) and antianginal drugs may have prognostic benefits in certain populations [4].

**Figure 1:**
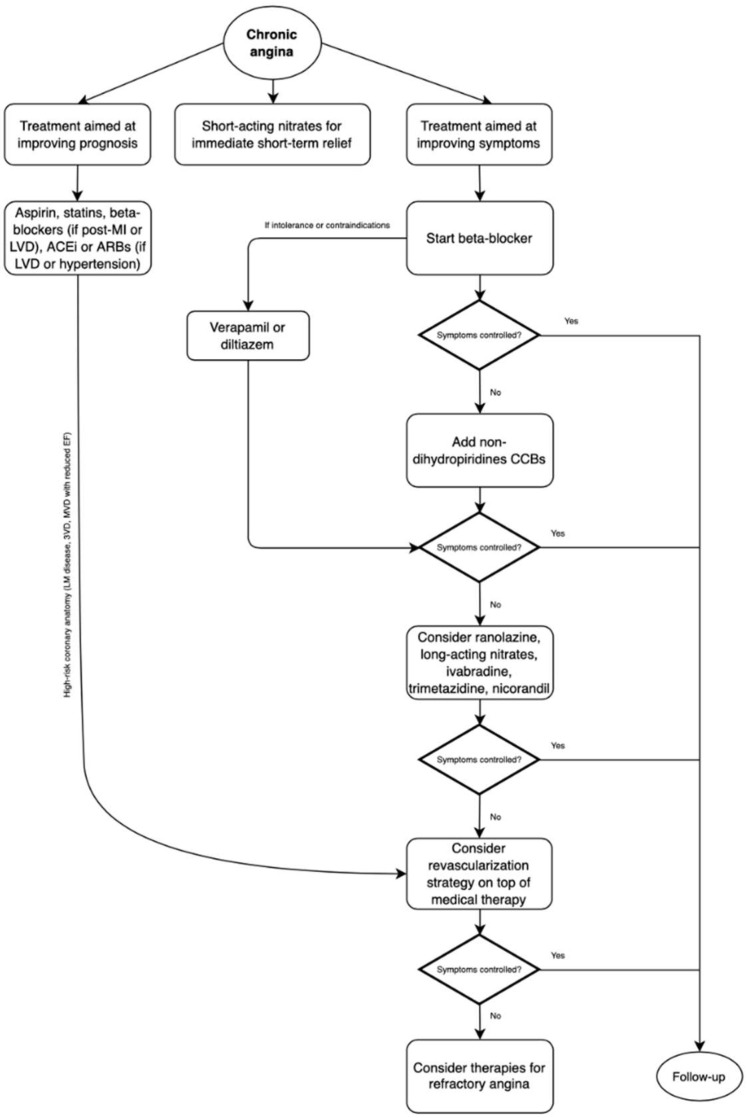
Management of chronic angina due to obstructive coronary artery disease. MI: myocardial infarction, LVD: left ventricular dysfunction, ACEi: angiotensin-converting enzyme inhibitors, ARBs: angiotensin-receptor blockers, CCBs: calcium channel blockers.

The management of chronic stable angina encompasses a broad spectrum of measures, from lifestyle modifications, various medications (which may affect prognosis and symptoms) and revascularization [4]. A stepwise approach for the management of chronic angina is proposed in Figure 1. 

## 3. Lifestyle Modifications

Healthy lifestyle behaviour should be encouraged in all patients, in particular regular exercise, healthy (i.e., Mediterranean) diet pattern, intentional weight loss and smoking cessation.

Cigarette smoking is responsible for 50% of deaths in smokers and a lifetime smoker has on average a 10-year life loss [9]. Smoking cessation improves the prognosis in patients with chronic coronary syndromes with a 36% reduction in mortality risk in quitters [10].

A Mediterranean diet, rich in fruit, vegetables, fibre, polyunsaturated fats, and legumes, with limited intake of red meat, processed foods, saturated fats and dairy is recommended in the European guidelines [11]. 

The lifetime risk of incident cardiovascular disease is higher in overweight and obese, and a healthy weight (BMI < 25) should be obtained and maintained, since intentional weight loss was associated with a lower risk of clinical outcomes in patients with CAD [12]. A physically active lifestyle (30–60 min moderate physical activity) should be encouraged, since it has been associated with lower mortality, with the lowest risk among those achieving high levels of activity [13].

## 4. Percutaneous Coronary Intervention and Coronary Artery Bypass Graft

Revascularization in stable ischemic heart disease has two main indications: to improve symptoms and to improve survival. In fact, in patients undergoing CABG for stable ischemic heart disease, survival benefits were observed only in the case of left main disease, triple vessel disease or ischemic cardiomyopathy [14,15,16,17]. The STICH trial randomized over 1200 patients with ischemic left ventricular dysfunction (LVEF < 35%) to receive medical therapy alone or CABG and medical therapy and showed the superiority of revascularization and medical therapy at 10-year follow-up for all-cause mortality, while the primary endpoint was not met at 5-year follow-up [14,15].

Patients with a left main disease have a high risk of adverse events and surgical revascularization demonstrated a 70% reduction in mortality during 5-year when compared to medical therapy [18].

It is noteworthy that most of these trials were conducted before the introduction of the most recent pharmacological agents for optimal medical therapy (OMT), such as intensive lipid-lowering therapy, dual antiplatelet therapy, heart failure medications and novel antidiabetic agents.

Similarly, no trial has shown a survival benefit of percutaneous coronary intervention over OMT in patients with stable angina [19,20,21]—neither if physiology guided [22]—even if a larger benefit could have been expected in subjects with high surgical risk, as those currently being treated for ischemic heart disease (IHD) [23,24].

The ISCHEMIA trial randomly assigned >5000 patients with moderate-to-severe ischemia (approximately 90% with a history of angina pectoris) to an initial invasive strategy with angiography and revascularization when feasible or to medical therapy alone and angiography if medical therapy failed. There was no significant difference between the two groups for the occurrence of ischemic cardiovascular events or death over a median of 3.2 years [21]; participants in the invasive strategy arm had larger improvements in angina-related health status than those randomized to conservative strategy arm, with minimal benefit in asymptomatic patients and larger benefits in those who had angina at baseline [25]. It is noteworthy that the trial excluded patients with recent acute coronary syndrome, unprotected left main disease > 50%, ejection fraction < 35%, NYHA class III or IV heart failure and unacceptable angina despite maximal medical therapy, therefore these results cannot be generalized to all chronic angina patients [21].

Furthermore, subgroup analysis of ISCHEMIA showed that neither moderate nor severe ischemia was associated with increased mortality relative to mild/no ischemia, while increasing CAD severity was associated with death and myocardial infarction for the most versus the least severe CAD subgroup. The most severe CAD subgroup had inferior rate of CV death or MI in the invasive strategy arm, with similar 4-year all-cause mortality [26].

The recent REVIVED-BCIS2 randomized 700 patients with ejection fraction ≤35%, extensive coronary artery disease suitable for PCI and demonstrable myocardial viability to receive PCI + OMT or OMT alone. Over a median of 41 months, there was no difference between the two groups for the primary outcome (death or heart failure hospitalization) and left ventricular ejection fraction, while quality-of-life scores appeared to favor the PCI group. However, since most patients had little or no angina at enrollment, these findings cannot be extrapolated to patients with moderate-to-severe angina and acute coronary syndromes [27].

Similarly, the management of the left main disease is still a matter of debate [28,29,30,31]. The NOBLE trial showed inferior clinical outcomes at 5-years follow-up with PCI when compared to CABG, mortality rates among groups were similar but higher rates of non-procedural MI and repeat revascularization were noticed in the PCI arm [29]. The EXCEL trial showed noninferiority of unprotected left main PCI in low-to-intermediate anatomic complexity (SYNTAX score <33) for the composite endpoint of death, stroke and myocardial infarction [31].

## 5. “Event-Reducing” Drugs

Medical therapies for cardiovascular risk reduction have the primary purpose of reducing the likelihood of a coronary event and its downstream complications (such heart failure, arrhythmias). This purpose may be achieved by stabilizing the atherosclerotic disease progression and reducing the response to thrombosis pathway [8]. 

Lipid-lowering therapies are a cornerstone in CAD managment and statins are usually the most prescribed drugs since their wide availability and solid evidence on cardiovascular adverse events reduction [32,33,34,35,36]. Adding ezetimibe to statin therapy resulted in further LDL cholesterol lowering and in a risk reduction of occurrence of the primary composite endpoint (CV death, nonfatal MI, unstable angina requiring rehospitalization, coronary revascularization or nonfatal stroke) on a median follow-up of 6 years [37]. Proprotein convertase subtilisin-kexin type 9 (PCSK-9) inhibitors alirocumab and evolocumab were both effective in reducing the risk of cardiovascular events in high-risk populations in the FOURIER and ODISSEY without significant safety concerns [38,39]. 

In patients with elevated triglyceride levels (135 to 499 mg/dl) despite the use of statins, icosapent ethyl significantly reduced the risk of ischemic events, including CV death, when compared to placebo [40]. Inclisiran and bempedoic acid have been proven effective in reducing LDL cholesterol of approximately 50% and 16%, respectively, when compared to placebo [41,42]. 

Antithrombotic therapy with aspirin is effective in reducing all-cause mortality in secondary prevention after ACS, while only combination of ASA + ticagrelor and ASA + clopidogrel + very low dose rivaroxaban were associated with better outcomes than aspirin alone (OR 0.8 for CV mortality in ASA + ticagrelor group, OR 0.64 for ASA + clopidogrel + rivaroxaban for all-cause mortality), with major bleeding increasing by 45–95% with dual antiplatelet therapy and 2–6 fold with triple antithrombotic therapy [43]. Some studies suggest use of clopidogrel over ASA for single antiplatelet therapy [44,45]. Dual antiplatelet therapy is recommended after PCI to reduce ischemic events in both chronic and acute coronary syndromes, even if its length may vary according to setting (ACS vs. CCS) and patient-specific ischemic (clinical and procedure-related factors) and bleeding risks [4,23,46,47]. In stable cardiovascular disease setting, low-dose rivaroxaban was associated with lower occurrence of the primary composite outcome (CV death, stroke, MI) and death from any cause than aspirin alone, at a cost of higher risk of major bleeding [48].

ACE-inhibitors (or angiotensin-receptor blockers in case of ACE-i intolerance) are recommended in patients with chronic coronary syndrome and coexisting hypertension, left ventricular dysfunction (LVEF ≤ 40%), chronic kidney disease or diabetes, while their benefit tend to disappear in the absence of heart failure or high cardiovascular risk [4,49,50,51].

Mineralocorticoid receptor antagonists such spironolactone are recommended in post-MI patients already on ACEi and beta-blocker with significant left ventricular dysfunction (LVEF ≤ 35%) and diabetes or heart failure [52]. Antidiabetic medications such GLP-1 receptor agonists and SGLT-2 inhibitors are increasingly used in cardiovascular disease since they reduce all-cause mortality, CV mortality, nonfatal myocardial infarction and kidney failure [4,5,23,53].

Colchicine has a broad anti-inflammatory effect, it may accumulate in neutrophils and macrophages and prevent the assembly of inflammasome and the expression of IL-1β, and proinflammatory cytokines [54]. Various RCTs showed its efficacy in reducing cardiovascular events in both chronic and acute coronary syndrome cohorts, but it is also noteworthy that some studies found a possible increase in non-CV deaths and in COPS trials (which did not meet the primary composite outcome in ACS patients) in the colchicine arm had higher all-cause mortality [55,56,57].

Further evidence on the negative effects of inflammation in the progression of cardiovascular disease is provided by the CANTOS trial, in which canakinumab showed efficacy in reducing the occurrence of the composite endpoint (nonfatal MI, nonfatal stroke, CV death) in patients with previous myocardial infarction and elevated CRP [58].

## 6. Symptomatic Drugs Not Conditioning Outcomes

### 6.1. Nitrovasodilators

Nitrates have various clinical indications such as angina pectoris, heart failure, and hypertensive emergencies and encompass organic nitrates (glyceryl trinitrate or nitroglycerin, isosorbide mononitrate, isosorbide trinitrate), sodium nitroprusside (direct nitric oxide donor) and nicorandil [59,60].

The characteristics of such compounds are summarized in Table 1.

Organic nitrates are prodrugs that need to be metabolized to release nitric oxide, which appears to be the effector of this class of compounds. Nitric oxide increases intracellular levels of cGMP. Such an increase in cGMP levels promotes dephosphorylation of the myosin light chain preventing interaction between actin and myosin and reducing cytosolic Ca^2+^, leading to smooth muscle cells relaxation in most tissues, including the arterial wall, leading to vasodilatation [61] and inhibition on platelet aggregation.

The effects of nitroglycerin as a remedy for angina pectoris are known since the end of the 19th century [62]. Low doses act predominantly on venous vessels, leaving the tone of resistance arterioles—and afterload—quite unaffected. This leads to a reduced venous return and decreases left and right ventricular preload, hence diminishing wall stress and cardiac oxygen demand. Furthermore, the reduction of left ventricular end-diastolic pressure allows better perfusion of subendocardial layers. However, even low doses of nitroglycerin produce vasodilation in large and medium coronary arteries and arterioles > 100 μm [63], face and neck arterioles, and meningeal vessels. In the absence of heart failure, organic nitrates cause a slight reduction of cardiac output, while preload reduction in the failing heart may induce beneficial effects on cardiac output.

Higher doses cause further vasodilation, reduced systemic blood pressure, and decrease systemic vascular resistances, which may activate compensatory sympathetic reflexed (e.g., tachycardia and positive inotropy) which increase oxygen consumption and potentially nullify the beneficial effects of coronary vasodilation.

Organic nitrates are rapidly absorbed from the skin, gastrointestinal tract, and mucous membranes [64].

Several preparations are available via different routes: transdermal patch/ointment, sublingual spray/tablets, buccal tablets, or intravenous formulations for hospitalized patients [59].

Short-acting nitrates are typically used as first-line therapy for acute effort angina since sublingual and spray GTN have a very rapid onset. Patients should be in a sitting position to avoid syncope. GTN may be also used for prophylaxis before physical activity to increase effort tolerance.

Long-acting nitrates are typically indicated when first-line therapy with beta-blocker or (non-dihydropyridine) calcium channel blocker is contraindicated, ineffective or poorly tolerated. 

Compared with placebo, transdermal nitroglycerin increase exercise duration without angina and total exercise time. It also decreases ST-segment depression after four hours, but not after 24 and 48 h [65]. Similar findings were noticed with a 24-h infusion of GTN, with rapid attenuation of benefits on exercise tolerance at the end of administration [66].

Denitrification of organic nitrates mediated by mitochondrial aldehyde dehydrogenase and ALDH-2 dysfunction is presumed to be the culprit of the mechanism of tolerance and loss of efficacy of nitrates [67].

Nitrate tolerance requires establishing nitrate-free intervals, in which angina may recur and exercise tolerance may be impaired, the so-called “zero-hour effect”; thus, abrupt withdrawal is not recommended and dosage should be tapered [59,68].

A common side effect of nitrate therapy is headache, which may be provoked by the hemodynamic effects of nitric oxide on the temporal artery [68,69]. Orthostatic hypotension, pallor, weakness, dizziness and light-headedness are also reported—usually with higher doses – while syncope is quite uncommon since hypotension is usually counterbalanced by reflex tachycardia. Beyond flushing and palpitations which are mild side effects, the occurrence of methemoglobinemia, although very rare and presumably dose-dependent, should be promptly recognized and treated accordingly [68,70].

Nitrates are contraindicated in patients with acute right ventricular infarction and hypertrophic cardiomyopathy with left ventricular outflow tract obstruction and should be used with extreme caution in volume-depleted patients and aortic stenosis. Co-administration with PDE-5 inhibitors and riociguat is contraindicated since the risk of hypotension, shock, and closed-angle glaucoma [4,68,71,72].

It is presumed that chronic therapy with nitroglycerin may increase free radical production by mitochondria and cause anomalies in mitochondrial respiration, thus worsening the endothelial dysfunction, and possibly increasing the vasoconstrictor sensitivity underlying rebound angina; long-term effects of these mechanisms are yet to be clarified [67,72].

Nicorandil has mixed vasodilator properties (both venous and arteriolar) due to its dual mechanism of action: nitric oxide donor and K^+^ ATP channel agonist. These effects cause a hyperpolarization of the vascular smooth cells and closure of L-type voltage-gated calcium channels, resulting in coronary and peripheral vasodilation [60,72]. Nicorandil is rapidly absorbed by the gastrointestinal tract and has a bioavailability >75%, reaches its peak plasma concentration in 30–60 min and has a half-life of 52 min. Its efficacy in stable angina was assessed in the SNAPE study, in which nicorandil seemed to be as safe and effective as isosorbide mononitrate in the elderly, even if isosorbide mononitrate may be superior for the symptomatic treatment of daily angina [73]. In the IONA trial, treatment with nicorandil in patients with stable angina was effective in reducing the primary composite endpoint (coronary heart disease death, nonfatal MI, unplanned admission for cardiac chest pain) [74].

Nicorandil is indicated as a second-line treatment for the management of chronic angina. 

Contraindications and side effects are common with organic nitrates, even if nicorandil can cause gastrointestinal, mucosal, cutaneous, or ocular ulceration, and should be immediately discontinued if any of these occurs. Data on the use of nicorandil and organic nitrates in pregnancy and breastfeeding are scarce and they should be avoided [72].

### 6.2. β-Blockers

Endogenous catecholamines are involved in the regulation of almost all tissues and systems and exert their effect through the adrenergic receptors.

At least three types of β receptors are known. The β_1_ receptors are primarily expressed in heart muscle and their activation result in increased chronotropy, inotropy and improved atrioventricular conduction. At the kidney level, activation of β_1_ receptors inhibits the release of renin from the juxtaglomerular cells, reducing the activity of the RAAs. β_2_ receptors are primarily expressed in peripheral vascular smooth muscle cells, bronchi, gastrointestinal tract, skeletal muscle, and uterus, while β_3_ in adipose tissue and heart and are implied in thermogenesis [75,76]. 

β-blockers are competitive ligands of G-protein and coupled β-receptors, inhibiting the effect of adrenaline and noradrenaline, decreasing the c-AMP synthesis and reducing protein-kinase-A activity. Such activity results in inhibition of L-type calcium channels, sarcoplasmic reticulum calcium/ATPase inhibitory protein and troponin-I, with negative inotropic and chronotropic effects and lower myocardial oxygen demand.

α_1_-adrenergic receptors are primarily expressed in venous, arterial, and visceral smooth muscle. 

α_2_-adrenergic receptors are responsible for the suppression of sympathetic stimulation and increase in vagal tone, inhibiting the release of neurotransmitters (acetylcholine and norepinephrine) and regulating metabolic pathways [75]. 

β-blockers have numerous differences among them, which are primarily affected by various properties, such as cardioselectivity (relative affinity for β_1_ and β_2_ receptors), intrinsic sympathomimetic activity, vasodilator capacity, differences in liposolubility, membrane-stabilizing activity and blockade of α-receptors [75].

The benefits of β-blockers in the treatment of effort angina are primarily due to a decrease in myocardial oxygen consumption at rest and during exertion, due to negative chronotropic and inotropic effects, with a reduction of arterial blood pressure [77]. Cardioselective β-blockers inhibit cardiac β_1_ receptors and have less effect on β_2_ receptors, at therapeutic doses, even if higher doses can also block β_2_ receptors being then preferred in the treatment of chronic angina. Some β-blockers exhibit partial agonist activity, which consists of β-agonist effect in case of low intrinsic adrenergic activity, and β-blocking activity in case of high intrinsic adrenergic activity. Principal agents of this subclass are acebutolol, pindolol and bucindolol.

Antiarrhythmic properties of carvedilol, metoprolol and propranolol rely, in addition to the common action of β-blockers, on sodium channels (like all class I antiarrhythmic drugs). 

Vasodilator β-blockers can modify vascular tone by blocking both α and β receptors (e.g., labetalol and carvedilol) or by nitric oxide-mediated vasodilatory action (e.g., nebivolol).

Lipophilic β-blockers have a short half-life and cross the blood-brain barrier and are extensively metabolized by the liver. Hydrophilic β-blockers have a longer half-life and renal clearance, whereas the capacity to cross the blood-brain barrier is inferior [75].

β-blockers may have various indications such as chronic angina, uncomplicated acute coronary syndrome, atrial and ventricular arrhythmias, systemic hypertension, heart failure, hypertrophic obstructive cardiomyopathy, thyrotoxicosis, migraine prophylaxis and variceal bleeding in portal hypertension.

Propranolol is the progenitor of β-blockers and has been proven effective for the treatment of angina pectoris. Treatment with propranolol is not associated with tachyphylaxis or an increased risk of death, although a possible increase in cardiogenic shock incidence was noted [78].

The positive effects of β-blockers have been documented in multiple trials [79,80,81]. Carvedilol proved effective in reducing blood pressure and heart rate and increasing coronary flow reserve in CAD patients. Atenolol reduced the risk for adverse outcomes in asymptomatic and mildly symptomatic patients with CAD compared with placebo. In some settings, the use of β-blockers may be particularly beneficial, e.g., post-myocardial infarction, heart failure, and supraventricular arrhythmias [82,83,84].

β-blockers are indicated as first-line therapy to control stable angina symptoms, their efficacy is like other antianginal drugs, with no significant evidence for improvement in survival (except for heart failure with reduced ejection fraction and post-MI) [85,86,87,88]. Characteristics are summarized in Table 2. 

β-blockers have negative chronotropic, dromotropic and inotropic effects. β-blockers should not be administered in patients with bradycardia, conduction disturbances or acutely decompensated heart failure [86]. They should also be avoided in vasospastic angina, severe peripheral vascular disease (especially nonselective ones, which may block β_2_ receptors and increase peripheral vascular resistance and thus worsen claudication), and reactive pulmonary obstructive diseases such as asthma and COPD. Caution should be used in pregnancy, low blood pressure, diabetes mellitus and Raynaud syndrome as well as during concomitant therapy with other negative chronotropic or dromotropic agents [4,75,86].

### 6.3. Calcium Channel Blockers

Calcium channels are expressed in various tissues, such heart, skeletal and smooth muscle, neurons, and spermatozoa. Different subtypes of Ca^2+^ channels exist, and the voltage-gated Ca^2+^ channels (L-type or slow channels) are predominant in the cardiovascular system and are responsible for the influx of extracellular calcium ions in smooth muscle, cardiomyocytes, and sinoatrial and atrioventricular nodal cells in response to electrical depolarization. T-type Ca^2+^ channels are also expressed in the heart, but they are expressed mostly in the embryonic phase and diminish during development, functioning as the pacemaker in the sinoatrial nodal cells of the adult heart [89]. Calcium is a trigger for the contraction of smooth muscle cells and cardiomyocytes. Calcium channel blockers bind to subunits of the transmembrane proteins reducing the frequency of channel opening when depolarization occurs, thus inhibiting calcium influx and leading to relaxation in vascular arterial beds and negative inotropic effects in the heart. The effects on smooth muscle cells and cardiac myocytes may vary according to the molecules.

Principally calcium channel blockers (CCBs) have various chemical structures, phenylalkylamines (verapamil), benzodiazepines (diltiazem), dihydropyridines (amlodipine, nifedipine, lercanidipine, nimodipine, felodipine, clevidipine, felodipine) and diarylaminopropylamines (bepridil).

CCBs have numerous different indications, such as angina pectoris, arrhythmias, systemic hypertension, Raynaud’s phenomenon, cerebral vasospasm, migraine, oesophagal spasm, and preterm labour [5,90].

All CCBs decrease arterial resistance, blood pressure and cardiac afterload, while the effect on veins and preload is not significantly affected. While dihydropyridine CCBs do not induce significant activation of the sympathetic nervous system, a possible increase in cardiac output and heart rate may be observed. Verapamil has direct negative inotropic, chronotropic and dromotropic effects and the sympathetic nervous system activation may be masked. The properties of diltiazem lie in between verapamil and dihydropyridines [66]. The positive effects of CCBs on angina may rely on the reduction of peripheral vascular resistance and left ventricular afterload, and, for both verapamil and diltiazem, in a direct reduction of contractility and oxygen demand [91]. In patients with variant angina, CCBs may exert a positive effect on coronary arterial tone [92]. The negative inotropic effect of dihydropyridines is neglectable at therapeutic doses. 

CCBs have different pharmacokinetics and oral bioavailability. Nifedipine has rapid absorption and produces a brief peak in plasma, thus determining blood pressure drop and reflex tachycardia, and possibly myocardial ischemia due to decreased coronary perfusion pressure [93]. Slow-release nifedipine produces less relevant fluctuations in plasma levels and is somehow more manageable. Amlodipine has slow absorption and a long half-life, thus producing minimal effects deriving from plasmatic fluctuations. 

Verapamil has various indications, including all types of angina (effort, vasospastic, unstable), supraventricular tachycardias, fascicular ventricular tachycardia, systemic hypertension, atrial fibrillation with rapid ventricular rate and claudication. It is also used to reduce left ventricular outflow tract obstruction in hypertrophic cardiomyopathy. 

In the Angina Prognosis Study in Stockholm (APSIS) study, verapamil was as effective as metoprolol in the management of angina pectoris, without a significant difference in cardiovascular mortality, and similar tolerability. A significant difference was found only for gastrointestinal side effects, which were more common with verapamil [94]. Compared with atenolol in hypertensive CAD patients, verapamil had similar efficacy but with lower angina frequency and fewer diagnoses of diabetes during follow-up [95].

Diltiazem has been proven effective in chronic stable angina, increasing exercise performance, time to onset of angina and ST-segment depression, with a favourable safety profile, compared with verapamil [75,96,97].

Long-acting nifedipine showed to be safe and effective in the treatment of angina, reducing the need for coronary angiography and intervention, with no effect on the rate of myocardial infarction or overall mortality [98].

Amlodipine demonstrated a significant dose-related increase in exercise duration and workload and a reduction in the number of anginal attacks and associated nitrate consumption. The antianginal effect of amlodipine was similar to the one of diltiazem and nadolol and resulted in fewer cardiovascular events at 24 months in the CAMELOT study. On a note, the CAMELOT enrolled patients with angiographically documented (>20%) CAD, also showing a slowing in atherosclerosis progression at IVUS assessment [99,100].

Non-dihydropyridines CCBs are indicated as an alternative to β-blockers, and dihydropyridines CCBs as an alternative or associated with β-blockers [4].

CCBs are extensively metabolized by the CYP3A4 group of cytochromes P-450 and verapamil inhibits the P-glycoprotein-mediated drug transport. Dihydropyridine CCBs do not inhibit the clearance of other substrates of cytochrome P-450 CYP3A, while verapamil and diltiazem do. Hence, caution should be used to avoid interactions with other substrates of CYP3A [90].

Since their cardioinhibitory effects, verapamil and diltiazem should not be used in patients with left ventricular dysfunction, bradycardia or conduction disturbances, and should be used with caution in the elderly and if associated with digitalis. Non-DHP CCBs should not be coadministered with β-blockers. Hypotension, severe aortic stenosis, and hypertrophic obstructive cardiomyopathy should preclude the use of dihydropyridine CCBs. Other adverse effects of CCBs include ankle swelling, cutaneous flush, palpitations, gingival growth, constipation, vertigo, and nausea [90,101].

### 6.4. Ranolazine

Ranolazine is an antianginal drug that selectively inhibits late Na^+^ current (I_NaL_) and a K^+^ current (I_Kr_). Inhibition of late Na^+^ current may reduce the sodium-dependent Ca^2+^ overload which is responsible for the contractile (diastolic tension increase), metabolic (less ATP synthesis) and electrophysiologic (arrhythmias) disturbances [102].

Ranolazine significantly reduces angina frequency and improves exercise duration and time to onset of angina [103]. Data on hard endpoints improvement in stable angina are lacking, and ranolazine nowadays has only data supporting its efficacy (except for one RCT [104]) in reducing symptoms [105,106], similarly to all other antianginal agents. 

Effects on heart rate and blood pressure are almost nil. Ranolazine has a particularly advantageous profile in diabetics since it demonstrated to significantly reduce HbA1c in both chronic angina and NSTE-acute coronary syndrome [106,107].

Some data support the use of ranolazine as an antiarrhythmic drug in atrial fibrillation [108], while data on coronary microvascular dysfunction are controversial, although European consensus contemplates ranolazine use for microvascular angina [6,102].

Ranolazine is contraindicated in severe hepatic or renal impairment, QT prolongation, and in association with CYP3A4 inhibitors and class Ia or III antiarrhythmic drugs (except amiodarone). Caution should be used in concomitancy with QT-prolonging drugs, CYP450 substrates or inhibitors, or glycoprotein-P inhibitors.

Frequent side effects are dizziness, nausea, headache, constipation, asthenia, bradycardia, blurry vision, QT prolongation, and peripheral oedema. Elderly people are more likely to experience side effects [102].

### 6.5. Ivabradine

Ivabradine exerts its negative chronotropic effect by antagonizing the transmembrane hyperpolarization-activated cyclic-nucleotide (HCN) gated channel, which controls the pacemaker current I_f_, a mixed Na^+^-K^+^ inward current which sets the rate of diastolic depolarization in the sinoatrial node, hence reducing the heart rate and myocardial oxygen demand, with no effects on inotropy [109].

Ivabradine is approved for the treatment of heart failure with reduced ejection fraction and stable angina pectoris in patients in which β-blockers are ineffective or not tolerated. Off-label uses for inappropriate sinus tachycardia and postural orthostatic tachycardia syndrome have been described.

In the INITIATIVE trial, ivabradine was as effective as atenolol in increasing total exercise duration and reducing the number of angina attacks [110].

In the SIGNIFY trial, the addition of ivabradine to standard care did not improve outcomes in a stable CAD population without clinical heart failure [111]. In the BEAUTIFUL trial, the reduction in heart rate with ivabradine resulted in an improvement of cardiac outcomes in patients with stable CAD and left ventricular systolic dysfunction, while it reduced hospital admissions for MI and coronary revascularization [112].

In chronic stable angina, ivabradine is indicated as second-line therapy and prescribed at a 5 mg bid dose, which can be increased to 7.5 mg bid if tolerated.

A meta-analysis showed that ivabradine was not effective in reducing cardiovascular-related mortality unless used for a specific indication, with a strong association with adverse events [113].

Common side effects are phosphenes, blurred vision, first-degree AVB, bradycardia, ventricular premature beats, nausea, dizziness, and atrial fibrillation [114].

Contraindications to ivabradine are resting heart rate < 70 bpm, acute coronary syndromes, high-degree atrioventricular block, atrial fibrillation, marked hypotension, acute heart failure or cardiogenic shock, severe hepatic impairment, and concomitant treatment with a potent CYP3A4 inhibitor. Women of childbearing age not using an adequate contraceptive should exert caution.

### 6.6. Trimetazidine

Trimetazidine partially inhibits long-chain 3-ketoacyl coenzyme-A-thiolase, the final enzyme in the free fatty acid β-oxidation pathway, leading to a partial shift from fatty acids to glucose oxidation in the heart. This process, although less profitable (fewer ATP produced), requires less oxygen and may be advantageous in ischemia [115].

Being a metabolic drug, trimetazidine does not affect inotropy or vasodilatory properties.

In a meta-analysis, trimetazidine reduced the number of weekly angina attacks, weekly GTN tablet consumption and improved exercise time to 1-mm ST-segment depression when compared with placebo [116].

Trimetazidine is indicated as second-line therapy in chronic angina and may be used in coronary microvascular dysfunction.

In diabetics, trimetazidine may improve HbA1c levels. In some patients, treatment with trimetazidine has been associated with Parkinsonian symptoms, restless leg syndrome, and gait instability, all reversible after discontinuation. Among side effects, nausea, hot flushes, diarrhoea, gastric or oesophagal burning, muscular cramps, depression, dizziness, sedation, visual disturbances, anorexia or hyperorexia were noticed.

Dose reduction may be required in those with renal impairment. Trimetazidine is contraindicated in severe renal impairment, Parkinson’s and motion disorders or pregnancy [117].

## 7. Angina with Non-Obstructed Coronary Arteries 

Angina with non-obstructed coronary arteries may encompass a wide spectrum of clinical presentations [118,119]. The two main mechanisms are coronary microvascular dysfunction and vasospastic angina. Microvascular angina is due to myocardial ischemia caused by coronary microvascular dysfunction, which may result from structural alteration or vasomotor disorders or both [120,121]. Vasospastic angina is due to dynamic epicardial coronary obstruction in a context of a vasomotor disorder [6,122].

Despite the absence of obstructive CAD, the prognosis, and quality of life of patients are affected negatively, especially if ischemia is documented by non-invasive functional tests [6].

In the diagnostic framework, invasive coronary angiography, measurement of left ventricular end-diastolic pressure, functional tests with adenosine and vasoreactivity test with acetylcholine are performed in an attempt to classify the patient in one of four categories: non-cardiac pain, epicardial vasospastic angina, microvascular angina, or a mixed form [120,123].

Information provided by left heart catheterization is of invaluable importance since management differs deeply. According to EAPCI consensus, patients with microvascular angina may be treated accordingly with β-blocker (e.g., nebivolol), dihydropyridines CCBs, ranolazine, trimetazidine, and ACEi/ARBs. VSA should be treated with CCBs (either DHP or non-DHP), nitrates or nicorandil. In mixed forms in which microvascular and vasospastic angina coexist, therapeutic options rely on CCBs, nicorandil, trimetazidine, ACEi/ARBs, and statins [6].

The recent EDIT-CMD trial randomized patients with angina with non-obstructive coronary arteries (ANOCA) to receive diltiazem or placebo. After 6 weeks, diltiazem therapy did not improve coronary vasomotor dysfunction, symptoms or quality of life, but did reduce the prevalence of epicardial spasm [92].

Equally important should be the prescription of drugs for risk factor management (hypertension, diabetes, dyslipidemia) and the advice to follow a healthy lifestyle [6,124].

Nowadays, ischemia with non-obstructed coronary arteries remains still an under-recognized and undertreated condition since the lack of uniform management and the lack of wide availability of invasive diagnostic tools [125].

## 8. Refractory Angina

The prevalence of refractory angina may vary between 5 and 10% of stable CAD patients. Refractory angina was defined as “a chronic condition characterized by the presence of angina caused by coronary insufficiency in the presence of coronary artery disease which cannot be controlled by a combination of medical therapy, angioplasty and coronary artery bypass surgery” [126].

Reasons to withhold further revascularization procedures are various and may range from intrinsic anatomic complexity of CAD with unsuitable anatomy to patient-related conditions with lack of graft material, poor LV function, severe extracardiac diseases, and advanced age.

Patients with refractory angina have a very poor quality of life with frequent hospitalizations, and therapeutic options, despite increasing, have often a low level of evidence in their favour [4].

Therapeutic nonpharmacological technologies in refractory angina address neural processing or myocardial perfusion (promoting collateral growth or transmurally redistributing blood flow). The ESC guidelines address with a low class of recommendation (IIb) enhanced external counterpulsation, coronary sinus reducer therapy and spinal cord stimulation to ameliorate symptoms and quality of life [4].

CD34+/CD133+ cell therapy is showing promising results, even in patients with coronary microvascular dysfunction, but it is still limited to research purposes [127]. Other therapies such as viral-transfer-based angiogenesis, transmyocardial laser revascularization, extracorporeal shockwave myocardial revascularization, and transcutaneous and subcutaneous electrical nerve stimulation lack solid placebo-controlled evidence [128].

## 9. Conclusions

**Figure 2:**
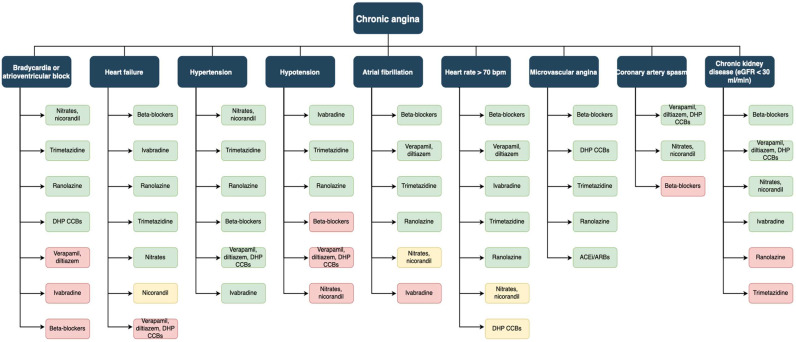
Management of chronic angina in different clinical settings. Adapted from Ferrari et al. [86]. Green boxes: recommendable or usable drugs. Yellow boxes: use with caution. Red boxes: generally contraindicated.

Despite numerous therapeutic innovations, patients with stable angina remain still a high-risk population. In particular, scarce control of angina symptoms leads to poor quality of life and increased hospitalizations. Numerous drugs have been demonstrated to improve prognosis and reduce major adverse cardiovascular events. Antianginal agents, on the other hand, improve quality of life with no clear evidence of prognostic improvement. Since their different actions and side effects, these drugs should be prescribed in a tailored fashion according to patient history and medications (Figure 2). 

In addition, patients affected by angina with non-obstructed coronary arteries and those with refractory angina constitute troublesome populations with the need for specific management and in which the evidence is still in progress. 

## Figures and Tables

**Table 1:** Characteristics of the principal nitrovasodilators in clinical practice. Adapted from Divakaran et al. [60].

Drug3.6	Route/Formulation	Dose	Time until Unset of Action	Duration of Action
**Nitroglycerin**	SL spray SL tablet TD patch IV infusion	0.4 mg 0.3–0.8 mg 0.2–0.8 mg/h 5–400 μg/kg/min	1–3 min 1–3 min 30 min Within seconds	25 min 25 min 10–12 h 3–5 min
**Isosorbide dinitrate**	PO IR tablet PO SR tablet	5–80 mg 40–160 mg	60 min 60 min	8 h 12 h
**Isosorbide mononitrate**	PO IR tablet PO SR tablet	5–20 mg 30–240 mg	30–45 min 30–45 min	6 h 12–24 h
**Sodium nitroprusside**	IV	0.3–10 μg/kg/min	1 min	6–12 min
**Nicorandil**	PO tablet	5–20 mg	30–60 min	12 h

**Table 2:** Properties and clinical use of principal β-blockers.

Drug.	Cardioselectivity	Membrane Stabilizing Activity	Alpha Blockage	Lipid Solubility	Indications
**Propranolol**	Nonselective	Yes	No	High	Angina, hypertension, ventricular arrhythmias, thyrotoxicosis, migraine prophylaxis, HOCM, variceal bleeding, essential tremor, pheochromocytoma
**Metoprolol**	Selective	Yes (high dose)	No	Moderate	Heart failure, ischemic heart disease, hypertension, arrhythmias, migraine prophylaxis, HOCM
**Nebivolol**	Selective	No	No	Low	Hypertension, heart failure
**Carvedilol**	Nonselective	Yes	Yes	Moderate	Heart failure, ischemic heart disease, hypertension
**Bisoprolol**	Selective	No	No	Low	Heart failure, ischemic heart disease, hypertension, arrhythmias
**Atenolol**	Selective	No	No	Low	Hypertension, ischemic heart disease, arrhythmias
**Labetalol**	Nonselective	Yes	Yes	Low	Chronic hypertension, hypertensive emergencies (eclampsia)
**Nadolol**	Nonselective	No	No	Low	Angina, hypertension, ventricular arrhythmias (LQTS, CPVT)

## Data Availability

Not applicable.
